# The spectrum of cardiac abnormalities in patients with acromegaly: results from a case-control cardiac magnetic resonance study

**DOI:** 10.1007/s11102-024-01403-1

**Published:** 2024-06-07

**Authors:** Dario De Alcubierre, Tiziana Feola, Alessia Cozzolino, Riccardo Pofi, Nicola Galea, Carlo Catalano, Renata Simona Auriemma, Rosa Pirchio, Rosario Pivonello, Andrea M. Isidori, Elisa Giannetta

**Affiliations:** 1 Department of Experimental Medicine, Sapienza University of Rome, Viale Regina Elena 324, Rome, 00161 Italy; 2 Neuroendocrinology, Neuromed Institute, IRCCS, Pozzilli, Italy; 3 Oxford Centre for Diabetes, Endocrinology, and Metabolism, Churchill hospital, Oxford University Hospitals, NHS Trust, Oxford, UK; 4 Department of Radiological Sciences, Oncology and Pathology, Sapienza University of Rome, Rome, Italy; 5 Dipartimento di Medicina Clinica e Chirurgia, Università Federico II di Napoli, Naples, Italy; 6 Centre for Rare Diseases (ENDO-ERN accredited), Policlinico Umberto I, Rome, Italy

**Keywords:** Acromegaly, Growth hormone, IGF1, Cardiomyopathy, Myocardial hypertrophy, Cardiac magnetic resonance

## Abstract

**Purpose:**

Cardiac abnormalities are common in patients with acromegaly, contributing to the increased morbidity and mortality. Cardiac magnetic resonance (CMR) is the gold standard for measuring cardiac morpho-functional changes. This study aims to detect cardiac alterations in acromegaly through CMR, even when the disease is adequately controlled.

**Methods:**

In this, multicentre, case-control study, we compared consecutive patients with acromegaly, cured after surgery or requiring medical treatment, with matched controls recruited among patients harbouring non-functioning adrenal incidentalomas.

**Results:**

We included 20 patients with acromegaly (7 females, mean age 50 years) and 17 controls. Indexed left ventricular-end-diastolic volume (LV-EDVi) and LV-end-systolic volume (LV-ESVi) were higher in patients than in controls (*p* < 0.001), as were left ventricular mass (LVMi) (*p* = 0.001) and LV-stroke volume (LV-SVi) (*p* = 0.028). Right ventricle (RV) EDVi and ESVi were higher, whereas RV-ejection fraction (RV-EF) was lower (*p* = 0.002) in patients than in controls (*p* < 0.001). No significant differences were observed in the prevalence of cardiometabolic comorbidities, including hypertension, glucose and lipid metabolism impairment, obstructive sleep apnoea syndrome, and obesity. IGF1 x upper limit of normal significantly predicted LVMi (b = 0.575; *p* = 0.008). Subgroup analysis showed higher LVMi (*p* = 0.025) and interventricular septum thickness (*p* = 0.003) in male than female patients, even after adjusting cardiac parameters for confounding factors.

**Conclusions:**

The CMR analysis reveals a cluster of biventricular structural and functional impairment in acromegaly, even when the biochemical control if achieved. These findings appear specifically triggered by the exposure to GH-IGF1 excess and show sex-related differences advocating a possible interaction with sex hormones in cardiac disease progression.

## Introduction

Acromegaly is associated with a typical cardiomyopathy, characterized by biventricular hypertrophy, mainly involving the left ventricle (LV), associated with diastolic dysfunction, in patients with active disease [1–4]; additional relevant cardiovascular complications include hypertension, valvopathies, arrhythmias, and vascular endothelial dysfunction, which, together with the respiratory and metabolic complications, contribute to the development of cardiovascular disease [1–3], mainly represented by atrial fibrillation and congestive heart failure [5].

Acromegaly is a slowly progressive disease characterized by increased release of growth hormone (GH) and, consequently, insulin-like growth factor I (IGF1), which is typically induced by a GH-secreting pituitary tumour [1]. Prolonged exposure to hormone excess leads to progressive somatic disfigurement and a wide range of systemic complications, such as cardiovascular, respiratory, and metabolic comorbidities, that are associated with increased morbidity and mortality [1–3].

The pathogenesis of acromegaly-related cardiomyopathy includes a direct action of GH and IGF1 excess on the heart, along with indirect mechanisms by which an excess of GH and IGF1 induces hypertension and glucose and lipid metabolism disorders, resulting in cardiac glucotoxicity and lipotoxicity with cardiac remodelling and hypertrophy [3, 6].

The duration of disease plays a pivotal role in the development of cardiomyopathy, because it is correlated with the prevalence of hypertension, diabetes mellitus and cardiac complications, suggesting a potential cumulative impact of long-term exposure to hormone excess [3]. Similarly, the age of acromegaly onset has been demonstrated to affect disease-related clinical outcomes [7].

Acromegaly control, induced by either pituitary surgery or medical therapy with conventional somatostatin analogs (SSAs) or pegvisomant (PEG), improves cardiac structure and performance [2, 3, 8–10], as well as vascular damage [3, 11], and haemodynamic and metabolic risk factors [12–15]. However, in a subset of patients, cardiovascular changes can persist even after remission, necessitating continuing management.

In this study, we aimed (i) to characterize cardiac alterations in patients with acromegaly using a cardiac magnetic resonance (CMR)-based approach, even when the disease is adequately controlled, and (ii) to identify potential clinical factors involved in the development of the cardiac dysfunction by matching the acromegaly cohort with randomly selected adrenal patients with proven intact hypothalamic-pituitary-adrenal-axis, but similar traditional cardiometabolic risk factors.

## Materials and methods

### Study population

This is a multicentre, case-control study involving consecutive male and female adult patients with acromegaly recruited from September 2014 to January 2020 from two Italian outpatient endocrinology clinics in the Department of Experimental Medicine at “Sapienza” University of Rome and the Department of Clinical Medicine and Surgery at “Federico II” University of Naples. Inclusion criteria were patients older than age 18 years with a previous diagnosis of acromegaly according to current guidelines [16], with a stable disease status for at least 6 months. Patients with acromegaly were defined as “cured” in case of persistent biochemical remission [i.e., nadir GH levels < 0.4 µg/L during oral glucose tolerance test, age and sex normalized IGF-1 or IGF1 per upper limit of normal (ULN) < 1.2] following pituitary adenomectomy; conversely, they were categorized as having “active disease” when showing persistently elevated IGF1 levels, thus requiring medical treatment to achieve biochemical control, including first or second generation of SSAs and/or PEG. Disease control during medical treatment was defined by age-sex normalized IGF-1 or IGF-1 levels < 1.2 x ULN and random GH levels < 1.0 µg/L, where applicable [17]. Exclusion criteria were contraindications to CMR; congenital or valvular cardiomyopathy, heart rhythm problems, congestive heart failure (NYHA II or III) or ischemic heart disease or revascularization after a myocardial infarction. The control group, matched for sex, age, and BMI, included subjects without acromegaly and without a history of cardiovascular disease, recruited among patients with non-functioning adrenal incidentaloma, undergoing follow-up imaging for the adrenal lesion at the Department of Experimental Medicine at “Sapienza” University of Rome. In each of these subjects, adrenal excess syndrome was ruled out after a full hormonal work-up, including morning serum cortisol levels following overnight 1 mg-dexamethasone, plasma renin and aldosterone levels, and 24 h urinary metanephrines, according to current criteria [18]. Twenty patients with acromegaly and 17 controls were enrolled. All subjects provided written informed consent to participate in the study. The study has been approved by the local Ethical Committee of Policlinico Umberto I (reference number 4244) and performed in accordance with the Declaration of Helsinki. This study adhered to the Strengthening the Reporting of Observational Studies in Epidemiology (STROBE) guidelines for reporting.

### Procedures

All subjects underwent an accurate history review, including hormonal assessment at diagnosis (IGF1 x ULN, GH, nadir GH during oral glucose tolerance test), physical examination, and blood sampling to assess glucose and lipid metabolism, and hormonal levels. Biochemical testing was performed locally at each centre. GH and IGF1 levels were analyzed by immunoradiometric and immunoenzymatic assays, depending on the different availability of each laboratory. All hormonal levels were analyzed in accordance with international standards. To better standardize results, IGF1 levels were normalized by sex- and age-adjusted ranges of each laboratory. All patients were referred to CMR imaging, which was performed in all cases by the same radiologist expert in cardiovascular imaging (N.G.) following the same protocol A 1.5-T scanner (Avanto, Siemens, Healthcare Solutions) with eight-element phased-array coil and electrocardiogram triggering was used. The standard CMR protocol was previously described [19].

### T1-mapping to evaluate fibrosis

We used the T1-mapping technique to non-invasively quantify the degree of myocardial fibrosis by measuring extracellular volume fraction (ECV), using the following formula: [DR1 myocardium: (1/T1 myocardial-post) - (1/T1 myocardial-pre); DR1 blood: (1/T1 blood-post) - (1/T1 blood-pre); Myocardial partition coefficient (λ) = (DR1 myocardial/DR1 blood); ECV = (1 - haematocrit) x (λ)] [20].

T1-mapping is automatically calculated as the average of the intensity of the individual pixels with and without contrast medium in T1 and expressed in milliseconds (CMR 42 SW).

### Statistical analysis

All continuous variables are expressed as mean and standard deviation (SD) or median with confidential interval [CI 5–95%], as appropriate. Dichotomous variables are expressed as frequencies and percentages when relevant. Student’s t test or the Mann-Whitney U test was performed to compare numerical variables between patients with acromegaly (ACRO) and controls (CTRLs), as well as within the ACRO cohort, after stratification based on a categorical variable. We performed one-way analysis of variance (ANOVA) or Kruskal-Wallis test to compare multiple groups of subjects according to type of medical treatment and disease status [i.e., cured, active disease], defined on current criteria [16, 21], and CTRLs. We carried out *post-hoc* analyses via Tukey’s or Dunn’s test, as appropriate, and results were reported only in case of statistically significant differences in the ANOVA and Kruskal-Wallis tests, respectively. One-way analysis of covariance (ANCOVA) or covariate-adjusted Kruskall-Wallis tests were performed to assess differences in continuous variables between active and cured patients, after controlling for covariates such as disease duration, sex and cardiometabolic comorbidities. We calculated mean estimates with 95% CI using Bonferroni corrections. Differences between qualitative variables were evaluated by χ2 statistics. We analysed correlations between numerical variables using Pearson’s or Spearman correlation test, as appropriate. Linear regression analysis was performed to evaluate whether parameters of disease activity at the time of diagnosis or CMR evaluation could predict cardiac impairment. We set the statistical significance at *p* < 0.05. Statistical analyses were performed using SPSS 20.0 for MacOS (SPSS Inc.).

## Results

### Patient characteristics

Twenty ACRO (7 females) with a mean age of 50 years (range 31–75 years) entered the study. The median time from diagnosis was 10 years (1–33). At the time of the original diagnosis, mean GH levels were 17.5 ± 12.1 and median IGF1xULN was 2.46 [1.08–4.94]. Eighteen (90%) had previously undergone trans-sphenoidal surgery. At the time of our evaluation, 6 patients (30%) were cured with surgery and 14 (60%) had active disease requiring medical treatment, including 5 patients on first-generation SSAs (25%), 4 on combined therapy with first-generation SSAs and PEG (20%), and 5 on pasireotide therapy (25%). At the time of study enrolment, 11 active patients (55%) exhibited disease control under medical treatment, only 3 (15%) presented with persistent biochemically uncontrolled disease. Median time between initial diagnosis and the achievement of disease control was 56.0 months (5-0-148.0).

### Biochemical and clinical evaluation

Table 1 compares the biochemical and clinical parameters (comorbidities and their therapies) of ACRO and CTRLs. As expected, sex, age, and BMI were similar between the two groups. We found no significant differences in fasting plasma glucose, HbA1c, lipid profile, blood pressure levels or the prevalence of cardiometabolic complications (hypertension, obesity, OSAS, dyslipidaemia, and glucose metabolism impairment) between ACRO and CTRLs, thus the two groups resulted homogeneous for cardiovascular and metabolic risk factors.

**Table 1:** Anthropometric, biochemical, clinical, and cardiac parameters at cardiac magnetic resonance (CMR) in patients with acromegaly and controls

	ACRO (n = 20)	CTRL (n = 17)	p value
Anthropometric parameters			
Sex (M/F)	13/7	8/9	0.457
Age, years	50.0 ± 12.4	57.5 ± 11.1	0.063
BMI kg/m^2^	27.6 (20.7–36.4)	26.5 (21.3–32.3)	0.456
Waist circumference, cm	101.5 (72.0-111.0)	100 (85.0-108.4)	0.503
Systolic BP, mmHg	120 (90–140)	120 (105–140)	0.477
Diastolic BP, mmHg	70 (58–90)	80 (60–85)	0.826
Heart rate, beats/min	64.5 ± 10.5	69.5 ± 9.1	0.127
Biochemical parameters			
Fasting plasma glucose, mg/dL	97 (63–124)	99 (77–143)	0.887
HbA1c, %	5.7 ± 0.66	5.8 ± 0.45	0.757
Fasting plasma insulin, µUI/mL	5.1 (1.5–16.0)	9.7 (3.4–26.8)	**0.015**
HOMA-i	1.05 (0.23–3.48)	2.08 (0.77–7.53)	**0.025**
Triglycerides, mg/dL	95.0 (42.0-231.0)	112.0 (38.0-216.8)	0.334
Total cholesterol, mg/dL	188.7 ± 34.3	198.4 ± 40.1	0.444
HDL cholesterol, mg/dL	53.5 ± 12.5	60.0 ± 11.0	0.328
LDL cholesterol, mg/dL	114.5 (63.0-181.0)	105.0 (50.0-179.4)	0.574
Clinical parameters			
OSAS, n (%)	2 (10.0%)	0 (0.0%)	0.081
Hypertension, n (%)	10 (50.0%)	7 (41.1%)	0.591
Obesity, n (%)	6 (30%)	4 (23.3%)	0.659
Diabetes mellitus, n (%)	3 (15.0%)	5 (29.4%)	0.289
IFG, n (%)	6 (30.0%)	3 (17.6%)	0.383
IGT, n (%)	1 (5.0%)	1 (5.8%)	0.906
Dyslipidemia, n (%)	9 (45.0%)	6 (35.2%)	0.549
Anti-hypertensive drugs	9 (45.0%)	7 (41.1%)	0.815
Anti-diabetic drugs	3 (15.0%)	4 (23.5%)	0.509
Lipid-lowering drugs	4 (20%)	4 (23.5%)	0.795
Cardiac parameters at CMR			
LV-EDVi (ml/m^2^)	79.6 ± 13.8	61.7 ± 11.5	**< 0.001**
LV-ESVi (ml/m^2^)	33.9 ± 7.8	23.8 ± 7.0	**< 0.001**
LV-SVi (ml/m^2^)	45.7 ± 9.2	37.9 ± 7.1	**0.007**
LV-EF (%)	57.5 ± 6.1	61.6 ± 6.7	0.069
LVMi (g/m^2^)	57.1 ± 14.2	43.5 ± 8.5	**0.001**
LVH (yes/no)/ (%)	1/20 (5.0%)	0/17 (0.0%)	0.350
Concentricity index (g/mL)	0.72 ± 0.15	0.74 ± 0.18	0.782
IVS thickness (mm)	11.5 (7.0–17.0)	10.0 (7.0-13.4)	0.128
RV-EDVi (ml/m^2^)	89.7 ± 19.7	63.4 ± 9.7	**< 0.001**
RV-ESVi (ml/m^2^)	44.8 ± 13.0	25.8 ± 6.4	**< 0.001**
RV-SVi(ml/m^2^)	44.9 ± 9.3	37.6 ± 7.6	**0.021**
RV-EF (%)	50.6 ± 6.0	59.3 ± 7.7	**0.001**
T1-preMean (ms)	995.5 ± 31.1	997.1 ± 18.7	0.889
T1-postMean (ms)	444.3 ± 51.3	406.9 ± 50.0	**0.038**
ECV (%)	26.0 ± 2.8	25.9 ± 2.1	0.979

Anthropometric clinical, biochemical, and morpho-structural and functional cardiac parameters in patients with acromegaly and matched controls at the time of enrolment.

M = male, F = female, BMI = body mass index, BP = blood pressure, OSAS = obstructive sleep apnea syndrome, IFG = impaired fasting glucose, IGT = impaired glucose tolerance

LV-EDVi = left ventricle end-diastolic volume indexed, LV-ESVi = left ventricle end-systolic volume indexed, LV-SVi = left ventricle stroke volume indexed, LV-EF = left ventricle ejection fraction, LVMi = left ventricle mass indexed, IVS = interventricular septum, RV-EDVi = right ventricle end-diastolic volume indexed, RV-ESVi = right ventricle end-systolic volume indexed, RV-SVi = right ventricle stroke volume indexed, RV-EF = right ventricle ejection fraction, ECV = extracellular volume

### Cardiac evaluation

**Fig. 1:**
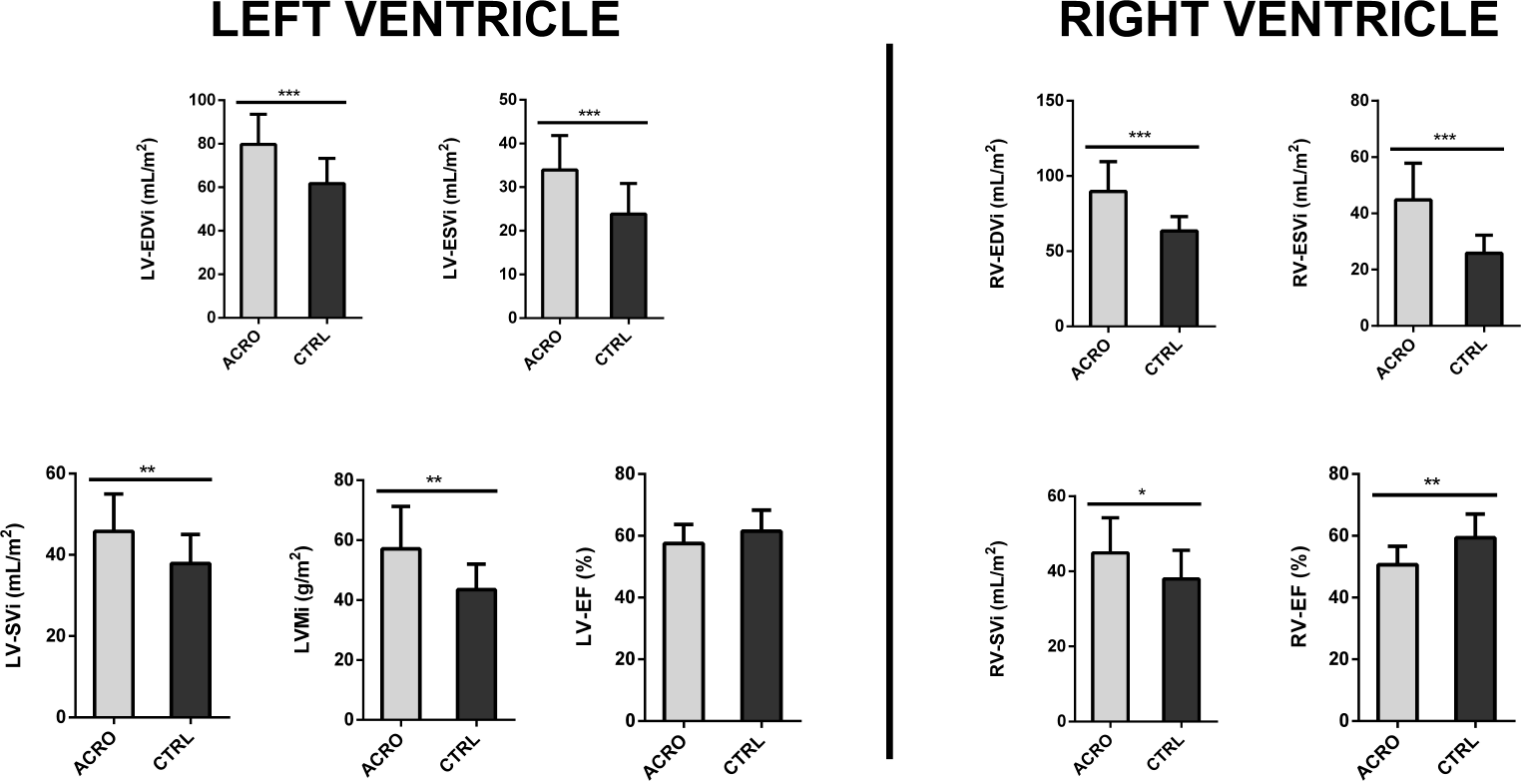
Cardiac morpho-structural and functional parameters in patients with acromegaly (grey bars, *n* = 20) and age, sex, and BMI-matched controls (black bars, *n* = 17). Data are expressed as mean ± SD. * = *p* < 0.05; ** = *p* < 0.01; *** = *p* < 0.001. ACRO = Acromegaly; CTRL = Controls; LV-EDVi = Left Ventricle End-Diastolic Volume index; LV-ESVi = Left Ventricle End-Systolic Volume index; LV-SVi = Left Ventricle Stroke Volume index; LVMi = Left Ventricular Mass index; LV-EF = Left Ventricle Ejection Fraction; RV-EDVi = Right Ventricle End-Diastolic Volume index; RV-ESVi = Right Ventricle End-Systolic Volume index; RV-SVi = Right Ventricle Stroke Volume index; RV-EF = Right Ventricle Ejection Fraction

Table 1; Fig. 1 summarize the cardiac parameters of ACRO and CTRLs. ACRO showed higher left ventricular (LV)-end diastolic volume index (EDVi) (79.6 ± 13.8 vs. 61.7 ± 11.5, *p* < 0.001), LV- end systolic volume index (ESVi) (33.9 ± 7.8 vs. 23.8 ± 7.0, *p* < 0.001) as well as left ventricular mass index (LVMi) (57.1 ± 14.2 vs. 43.5 ± 8.5, *p* = 0.001). Consequently, LV stroke volume index (LV-SVi) was higher in ACRO than in CTRLs (45.7 ± 9.2 vs. 37.9 ± 7.1, *p* = 0.007), whereas a trend was observed towards lower LV- ejection fraction (EF) in ACRO (57.5 ± 6.1 vs. 61.6 ± 6.7, *p* = 0.069). Concentricity index (LVMi/LV-EDVi) and interventricular septum (IVS) thickness did not significantly differ between the two groups (*p* = 0.782 and *p* = 0.128, respectively). Only one ACRO had LV hypertrophy (LVH) according to conventional CMR thresholds [22, 23]. Specifically, said patient was diagnosed with acromegaly at age 32; following marked changes in facial and body features, the diagnostic workup revealed severe acromegaly (IGF1xULN at diagnosis: 3.94) caused by a GH-secreting, locally invasive pituitary macroadenoma (maximum lesion diameter: 19 mm). The patient underwent pituitary adenomectomy and subsequently received SSA treatment due to persistent disease activity. Of note, the patient was treated with increasing doses of SSAs, but had not yet achieved disease control at the time of study enrolment, two years after medical treatment initiation (GH 2.1 ng/ml, IGF1xULN 1.6). Differences in right-ventricular (RV) parameters between groups resembled the ones observed in LV parameters. RV-EDVi and RV-ESVi were higher in ACRO than CTRLs (89.7 ± 19.7 vs. 63.4 ± 9.7 and 44.8 ± 13.0 vs. 25.8 ± 6.4, respectively; *p* < 0.001 for both). RV-SVi was also higher in ACRO than in CTRLs (44.9 ± 9.3vs 37.6 ± 7.6, *p* = 0.021). Moreover, ACRO showed a lower RV-EF than CTRLs (50.6 ± 6.0 vs. 59.3 ± 7.7, *p* = 0.001). Evaluation with T1 mapping technique did not reveal any significant difference between ACRO and CTRLs prior to administration of intravenous contrast; conversely, we found post contrast T1 intensity to be higher in ACRO than in CTRLs (444.3 ± 51.3 vs. 406.9 ± 50.0, *p* = 0.038). Three patients (2 with controlled disease under medical treatment and 1 surgically cured) had ECV greater than 30%, indicating the presence of myocardial fibrosis [24]; however, ECV did not significantly differ between the two groups (*p* = 0.979).

**Fig. 2:**
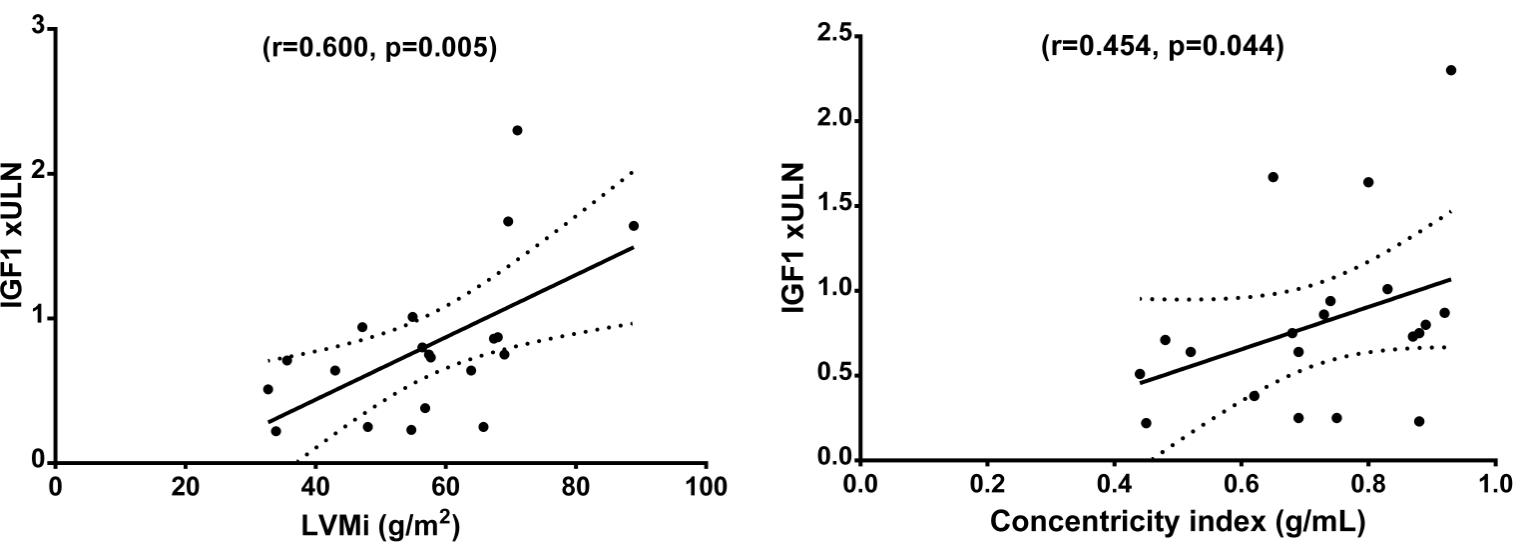
Scatterplot depicting the correlations between IGF1 levels times the upper limit of normal and cardiac parameters. IGF-1 x ULN = insulin-like growth factor-1 times the upper limit of normal; LVMi = Left Ventricular Mass index. The cut-off used to define left hypertrophy was LVMi higher than 83 g/m^2^ in men and 67 g/m^2^ in women. The upper limit of normal for concentricity index is defined as 0.9 g/mL in men and 0.8 g/mL in women

We found a significant correlation between IGF1 x ULN and LVMi (*r* = 0.600; *p* = 0.005), and concentricity index (*r* = 0.454; *p* = 0.044), and linear regression analysis confirmed IGF1 x ULN as a predictor for LVMi (B = 0.575, *p* = 0.008) in the ACRO group, see Fig. 2. Conversely, neither hormone levels at diagnosis (i.e., mean GH, IGF1xULN) nor duration of biochemical uncontrolled disease showed significant associations with any of the cardiac parameters at baseline.

### Subgroup analysis according to cardiometabolic comorbidities, disease duration and sex

To investigate the potential impact of cardiometabolic impairment on cardiac structure and function, we compared ACRO based on the presence or absence of several comorbidities and risk factors, including hypertension, obesity, diabetes mellitus, impaired fasting glucose, impaired glucose tolerance, OSAS, dyslipidaemia, and smoking. Notably, the 3 patients with diabetes (2 on pasireotide, 1 on combined SSA and PEG treatment) showed lower LV-EF compared to those without diabetes (50.2 ± 2.0 vs. 58.8 ± 5.7, *p* = 0.021), whereas we observed no significant differences according to the other factors.

Furthermore, we tried to ascertain the impact of disease duration on cardiac dysfunction. The median disease duration, measured in years passed since the initial diagnosis, was 10 years (1–33). Of note, the duration of disease was similar between active and cured patients, and it was not associated with any of the main cardiac parameters.

Lastly, a comparison according to sex revealed that male patients with acromegaly exhibited higher BMI compared to females (23.0 [20.7–27.8] vs. 29.0 [24.0-38.4], *p* = 0.007) without other significant differences in cardiometabolic risk factors. Male patients with acromegaly presented with higher LV-ESVi (37.0 ± 7.0 vs. 28.0 ± 5.9, *p* = 0.011), LVMi (65.1 ± 9.1 vs. 42.1 ± 8.3, *p* < 0.001), IVS thickness (13.0 [9.0–17.0] vs. 9.0 [7.0–13.0], *p* = 0.002), RV-EDVi (96.1 ± 20.2 vs. 77.8 ± 12.5, *p* = 0.044), RV-ESVi (49.8 ± 12.6 vs. 35.3 ± 7.3, *p* = 0.013), postcontrast T1 intensity (471.2 ± 46.5 vs. 394.0 ± 28.1, *p* = 0.002) and lower RV-EF (48.4 ± 5.9 vs. 54.6 ± 4.1, *p* = 0.023) compared to females. After adjusting cardiac parameters for population age- and sex- reference ranges, male patients confirmed higher LVMi (*p* = 0.025) and IVS thickness (*p* = 0.003) than females.

### Subgroup analysis according to disease status

To evaluate the impact of disease status on cardiac dysfunction, we compared cardiac parameters between controls, patients with active disease on medical treatment and those in long-term biochemical remission following surgery.

**Fig. 3:**
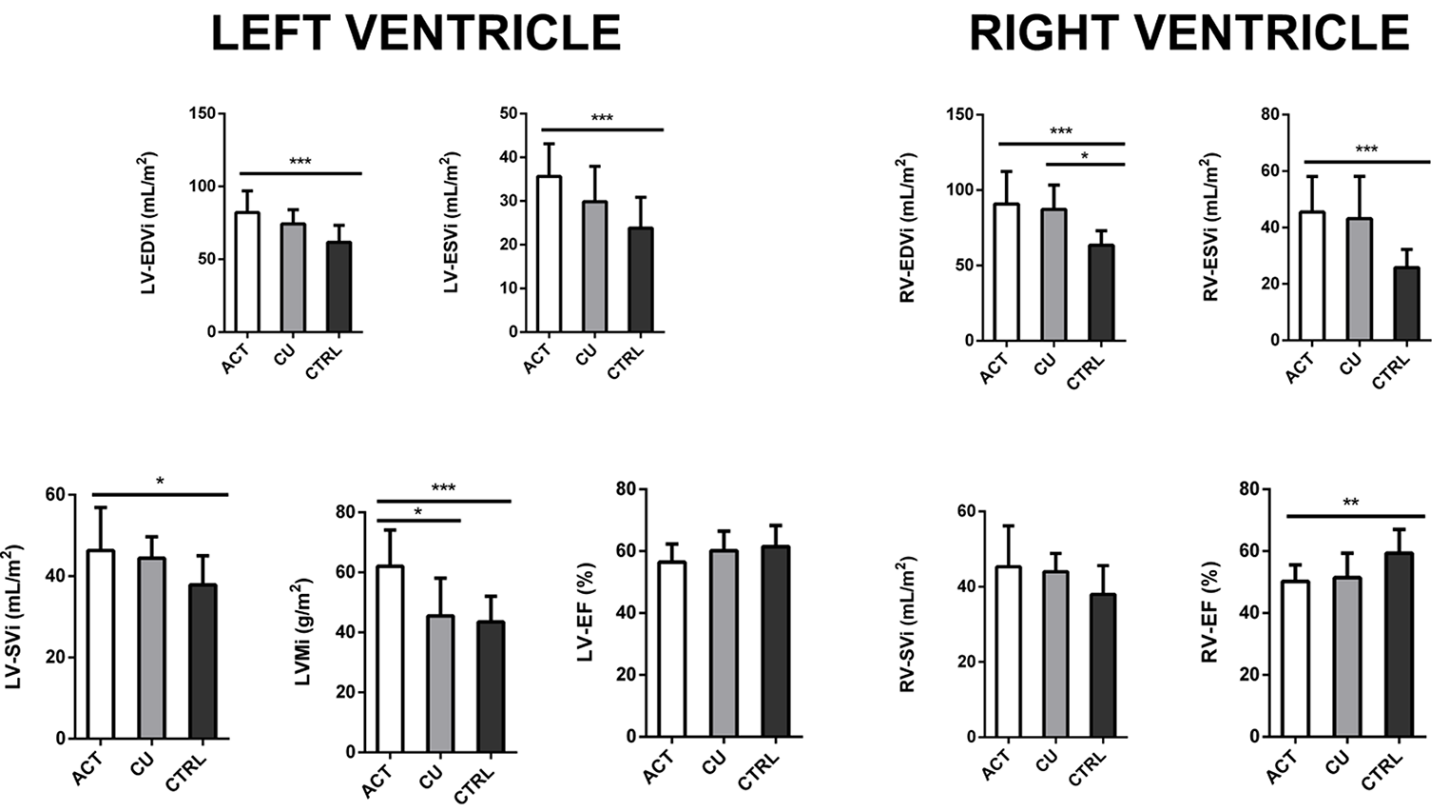
The main cardiac morpho-structural and functional parameters in patients with acromegaly after stratification for disease status. Comparisons were made among patients with active acromegaly (white bars, *n* = 14), patients in remission from acromegaly after surgery (grey bars, *n* = 6) and age, sex, and BMI-matched controls (black bars, *n* = 17). Data are expressed as mean ± SD. * = *p* < 0.05; ** = *p* < 0.01; *** = *p* < 0.001. LV-EDVi = Left Ventricle End-Diastolic Volume index; LV-ESVi = Left Ventricle End-Systolic Volume index; LV-SVi = Left Ventricle Stroke Volume index; LVMi = Left Ventricular Mass index; LV-EF = Left Ventricle Ejection Fraction; RV-EDVi = Right Ventricle End-Diastolic Volume index; RV-ESVi = Right Ventricle End-Systolic Volume index; RV-SVi = Right Ventricle Stroke Volume index; RV-EF = Right Ventricle Ejection Fraction

Table 2 and Fig. 3 summarize the results of post-hoc analyses.

**Table 2:** Subgroup analysis of morpho-structural and functional cardiac parameters at cardiac magnetic resonance according to disease status

	Controls (*n* = 17)	Active ^a^ (*n* = 14)	Cured ^b^ (*n* = 6)	*P*^c^ value	*P*^d^ value	*P*^e^ value
Sex (M/F)	8/9	10/4	3/3	0.461	1.000	0.202
Age, years	57 ± 11	50 ± 14	49 ± 8	0.232	0.335	0.986
Years from diagnosis	NA	8 (2.0-24.8)	10 (1.7–31.7)	-	-	0.552
Time until disease control (months)	NA	48.5 (7.0-216.0)	56.0 (5.0-148.0)	-	-	0.754
GH at diagnosis	NA	16.3 ± 12.7	17.9 ± 12.6	-	-	0.509
IGF1 xULN at diagnosis	NA	2.66 [1.34–5.92]	1.69 [0.82–2.03]	-	-	0.076
IGF1 xULN at baseline	NA	0.80 (0.54–1.16)	0.62 (0.34–0.74)	-	-	0.130
LV-EDVi (ml/m^2^)	61.7 ± 11.5	82.0 ± 14.9*	74.3 ± 9.8	< 0.001	0.110	0.438
LV-ESVi (ml/m^2^)	23.8 ± 7.0	35.6 ± 7.4*	29.8 ± 8.0	< 0.001	0.206	0.252
LV-SVi (ml/m^2^)	37.9 ± 7.1	46.3 ± 10.5^§^	44.4 ± 5.2	0.023	0.243	0.889
LV-EF (%)	61.5 ± 6.7	56.4 ± 5.9	60.1 ± 6.3	0.083	0.894	0.462
LVMi (g/m^2^)	43.5 ± 8.5	62.0 ± 12.1*	45.5 ± 12.5^#^	< 0.001	0.915	0.009
Concentricity index (g/mL)	0.73 ± 0.19	0.76 ± 0.11	0.62 ± 0.20	0.905	0.343	0.222
RV-EDVi (ml/m^2^)	63.4 ± 9.7	90.8 ± 21.5*	87.1 ± 16.1^§^	< 0.001	0.012	0.887
RV-ESVi (ml/m^2^)	25.8 ± 6.4	45.5 ± 12.6*	43.1 ± 15.0	< 0.001	0.005	0.894
RV-SVi(ml/m^2^)	37.9 ± 7.6	45.3 ± 10.8	44.0 ± 4.8	0.069	0.329	0.949
RV-EF (%)	59.3 ± 7.7	50.2 ± 5.3*	51.4 ± 7.9	0.003	0.058	0.931
T1-preMean (ms)	997.1 ± 18.7	991.8 ± 33.8	1005 ± 21.5	0.899	0.844	0.610
T1-postMean (ms)	408.8 ± 42.9	446.9 ± 54.1	446.6 ± 63.9	0.106	0.317	1.000
ECV (%)	25.9 ± 2.1	26.1 ± 2.6	25.6 ± 3.4	0.981	0.966	0.922

Morpho-structural and functional cardiac parameters in matched controls and patients with acromegaly, after stratification according to disease status

^a^ Patients were defined as “active” in case of persistently elevated IGF1 levels, thus requiring medical treatment for acromegaly

^b^ Patients were defined as “cured” in case of persistent biochemical remission [i.e., nadir GH levels < 0.4 µg/L during oral glucose tolerance test, IGF1 per upper limit of normal (ULN) < 1] following pituitary adenomectomy

^c^ Healthy controls vs. patients with active acromegaly

^d^ Healthy controls vs. patients cured from acromegaly

^e^ Hatients cured from acromegaly vs. patients with active acromegaly

M = male, F = female, NA = not applicable, LV-EDVi = left ventricle end-diastolic volume indexed, LV-ESVi = left ventricle end-systolic volume indexed, LV-SVi = left ventricle stroke volume indexed, LV-EF = left ventricle ejection fraction, LVMi = left ventricle mass indexed, IVS = interventricular septum, RV-EDVi = right ventricle end-diastolic volume indexed, RV-ESVi = right ventricle end-systolic volume indexed, RV-SVi = right ventricle stroke volume indexed, RV-EF = right ventricle ejection fraction, ECV = extracellular volume

The results showed higher LVMi in patients with active disease compared to the ones who had been cured with surgery (62.0 ± 12.0 vs. 45.5 ± 12.5, *p* = 0.013), even after correcting for sex (*p* = 0.011), whereas no differences in the remaining cardiac parameters were detected.

Patients with active disease also presented with higher LV-EDVi (82.0 ± 14.9 vs. 61.7 ± 11.5, *p* < 0.001), LV-ESVi (35.6 ± 7.4 vs. 23.8 ± 7.0, *p* < 0.001), LV-SVi (46.3 ± 10.5 vs. 37.9 ± 7.1, *p* = 0.023), LVMi (62.0 ± 12.1 vs. 43.5 ± 8.5, *p* < 0.001) compared to healthy controls, even after correcting for sex (*p* < 0.001).

On the other hand, left ventricle morphologic parameters were similar between controls and patients surgically cured from acromegaly (Table 2).

Regarding the RV, controls exhibited lower RV-EDVi compared to acromegaly patients, both with active disease (63.4 ± 9.7 vs. 90.8 ± 21.5, *p* < 0.001) and in biochemical remission after surgery (63.4 ± 9.7 vs. 87.1 ± 16.1, *p* = 0.012), even after correcting for sex (*p* < 0.001, *p* = 0.010). Furthermore, controls showed lower RV-ESVi compared to patients with active disease (25.8 ± 6.4, vs. 45.5 ± 12.6 *p* < 0.001), even after correcting for sex (*p* < 0.001); higher RV-EF compared to patients with active acromegaly (59.3 ± 7.7 vs. 50.2 ± 5.3, *p* = 0.003) and cured patients (59.3 ± 7.7 vs. 51.4 ± 7.9, *p* = 0.058), even after correcting for sex (*p* = 0.005; *p* = 0.040).

Lastly, as shown in Table 2, active and cured patients showed a comparable hormonal profile at diagnosis regarding mean GH levels (*p* = 0.509), with only a trend towards higher IGFxULN levels (*p* = 0.076) in patients with active disease at the time of enrolment. Time between initial diagnosis and the achievement of disease control (either via medical therapy or persistent remission following surgery) was also comparable between groups (*p* = 0.754).

### Subgroup analysis according to type of medical treatment

Lastly, to assess potential differences in cardiac morphology and function induced by various medical treatments, we categorized patients with active disease based on their medication regimen at time of study enrolment. Specifically, we compared patients receiving first-generation SSAs (*n* = 5), second-generation SSA pasireotide (*n* = 5), and combined treatment with first-generation SSAs and PEG (*n* = 4). Table 3 detailed the results of this subgroup analysis. In summary, we did not detect any significant differences in the main disease-related and cardiac variables among the three treatment groups, except for a lower RV-EF in patients on combined medical treatment (*p* = 0.023).

**Table 3:** Subgroup analysis of morpho-structural and functional cardiac parameters at cardiac magnetic resonance according to type of medical treatment

	SSA (*n* = 5)	PASI (*n* = 5)	SSA + PEG (*n* = 4)	*P*^a^ value	*P*^b^ value	*P*^c^ value
Sex (M/F)	4/1	2/3	4/0	0.197	0.343	0.167
Age, years	55.6 ± 21.2	44.8 ± 9.2	50.5 ± 7.8	0.492	0.862	0.831
Years from diagnosis	6.0 (2.2–16.7)	8.2 (2.5–16)	15.2 (11.2–19)	0.978	0.314	0.403
GH at diagnosis	21.6 ± 18.1	14.8 ± 10.2	48.6 ± 27,4	0.956	0.522	0.375
IGF1xULN at diagnosis	3.03 (2.6–6.4)	2.01 (1.5–3.7)	2.09 (1.3–2.8)	0.359	0.397	0.981
IGF1 x ULN at baseline	0.94 (0.56–1.97)	0.64 (0.24–1.21)	0.80 (0.66–0.97)	0.423	0.615	0.962
LV-EDVi (ml/m^2^)	82.4 ± 17.9	80.1 ± 16.7	83.7 ± 12.3	0.971	0.992	0.940
LV-ESVi (ml/m^2^)	36.7 ± 8.0	32.4 ± 8.7	38.4 ± 4.7	0.652	0.941	0.487
LV-SVi (ml/m^2^)	45.7 ± 10.2	47.6 ± 13.7	45.3 ± 9.3	0.963	0.998	0.951
LV-EF (%)	55.4 ± 2.0	59 − 4 ± 8.7	53.8 ± 4.3	0.557	0.916	0.374
LVMi (g/m^2^)	68.1 ± 14.8	56.8 ± 12.1	60.9 ± 5.7	0.329	0.646	0.871
Concentricity index (g/mL)	0.82 ± 0.09	0.72 ± 0.15	0.73 ± 0.06	0.334	0.447	0.989
RV-EDVi (ml/m^2^)	84.3 ± 24.6	90.8 ± 23.4	98.9 ± 17,8	0.892	0.612	0.856
RV-ESVi (ml/m^2^)	39.3 ± 12.3	44.4 ± 13.0	54.5 ± 9.1	0.774	0.177	0.437
RV-SVi(ml/m^2^)	45.0 ± 13.5	46.2 ± 11.3	44.4 ± 9.6	0.980	0.997	0.965
RV-EF (%)	53.5 ± 4.6	51.3 ± 4.2	44.7 ± 3.0	0.692	**0.021**	0.081
T1-preMean (ms)	994.2 ± 26.1	1004.6 ± 39.3	973.0 ± 34.9	0.879	0.631	0.378
T1-postMean (ms)	449.4 ± 55,1	430.8 ± 63.6	464.0 ± 54.4	0.865	0.923	0.669
ECV (%)	26.7 ± 3.4	26.9 ± 2.9	24.6 ± 1.1	0.996	0.541	0.459

Morpho-structural and functional cardiac parameters at cardiac magnetic resonance in patients with active acromegaly, after stratification according to type of medical treatment at the time of enrolment. Patients were defined as “active” in case of persistently elevated IGF1 levels, thus requiring chronic medical treatment for acromegaly

SSA = first-generation somatostating analogs, PASI = pasireotide, SSA + PEG = combined treatment with SSAs and pegvisomant, M = male, F = female, LV-EDVi = left ventricle end-diastolic volume indexed, LV-ESVi = left ventricle end-systolic volume indexed, LV-SVi = left ventricle stroke volume indexed, LV-EF = left ventricle ejection fraction, LVMi = left ventricle mass indexed, IVS = interventricular septum, RV-EDVi = right ventricle end-diastolic volume indexed, RV-ESVi = right ventricle end-systolic volume indexed, RV-SVi = right ventricle stroke volume indexed, RV-EF = right ventricle ejection fraction, ECV = extracellular volume

^a^ Patients on first-generation SSAs vs. patients on pasireotide

^b^ Patients on first-generation SSAs vs. patients on combined SSA and PEG treatment

^c^ Patients on pasireotide vs. patients on combined SSA and PEG treatment

## Discussion

The current multicentre, case-control study shows CMR features of biventricular impairment in acromegaly, even when the biochemical control if achieved, consisting of (i) higher biventricular volumes; (ii) higher LV mass and (iii) lower RV systolic performance compared with sex, age, and BMI-matched CTRLs with a similar cardiometabolic risk profile. These results suggest a predominant role of the exposure to GH-IGF1 excess on the heart, which also appears to be more detrimental in patients with active disease requiring medical treatment compared to those surgically cured, and in men compared to women.

Due to its higher accuracy and reproducibility and lower variability in comparison with echocardiography, CMR is the established non-invasive gold standard method for measuring cardiac morpho-functional changes [25], although its use might be limited by its cost and its limited access. To date, few studies have analysed patients with acromegaly using CMR [26–31], only two of which have compared patients with healthy matched controls, showing higher cardiac mass and volumes in acromegaly, without changes in cardiac function [10, 28]. Our results confirm these findings. However, our study uniquely compared patients with acromegaly with controls with the same cardiometabolic risk factors, thereby highlighting the direct role of GH-IGF1 excess, independently of traditional comorbidities (e.g. hypertension and diabetes mellitus).

Although the majority of the CMR studies found higher LVMi, the percentage of LVH varied among different studies, ranging from 72% to 5–8% [10, 26, 29–31]. In our cohort, the prevalence of LVH was 5% (1/20 patients), in line with the latest studies using the same cut-off for LVH [29, 30].

Recent CMR studies found associations between LVMi and various clinical factors, such as age, sex BMI, disease duration, hypertension, and hormonal levels [10, 31]. In our cohort, we observed a significant correlation between IGF1 x ULN and both LVMi and the concentricity index; conversely, we did not find any correlations with either disease duration or the time interval between initial diagnosis and disease control attainment. Similarly, our data showed that, in the overall acromegaly cohort, hormonal levels at diagnosis were not associated with worse cardiac outcomes at the time of CMR analysis; nevertheless, we observed a trend towards higher IGF1xULN levels at diagnosis in patients with active disease compared to the ones in remission at the time of study enrolment: although this difference did not reach statistical significance, likely due to the small sample size, these results would suggest a potential impact of pre-diagnosis disease severity on the development of cardiac abnormalities. The results of regression analysis support a direct impact of IGF1 levels on cardiac structure in patients with acromegaly, underlining the importance of disease control in these patients. Indeed, the subgroup analysis according to disease status confirmed that active patients showed worse cardiac parameters than controls and higher LVMi than patients surgically cured.

Evidence collected in animal and human models support the role of GH and IGF1 in determining direct changes in cardiac muscle. GH and IGF1 receptors are expressed, and IGF1 is synthesized directly in cardiomyocytes. In animal models, GH and IGF1 increase myocardial contractility and induce a hypertrophic response of the heart and GH stimulates cardiac myocytes to re-enter the cell cycle, increasing the number of cardiac myocytes [3].

However, the pathogenesis of acromegaly-related cardiomyopathy includes both a direct action of GH and IGF1 excess on the myocardium and indirect mechanisms induced by hormone excess, such as hypertension and derangements in glucose and lipid metabolism; these effects result in cardiac glucotoxicity and lipotoxicity, leading to cardiac remodelling and hypertrophy. In our cohort, 50% of patients with acromegaly presented hypertension and this could have contributed to cardiac impairment. Moreover, the presence of other acromegaly-related cardiovascular risk factors, such as glucose metabolism impairment (45% of patients), OSAS (10%) and dyslipidemia (45% of patients), might also have had a role. Indeed, our subgroup analysis showed that patients with diabetes and acromegaly had poorer systolic function as compared with patients without diabetes. It is known that pasireotide treatment can lead to hyperglycaemia and impaired insulin secretion; hence, when observing cardiac abnormalities in patients undergoing pasireotide treatment, the causative factor may predominantly be diabetes rather than acromegaly. Noteworthy, in our cohort, 2/5 pasireotide-treated patients presented with diabetes mellitus. We performed a subgroup analysis according to medical treatment at the time of study enrolment, that did not show a worse cardiac profile in pasireotide-treated patients compared to those treated with either SSAs or SSAs and PEG.

Ventricular hypertrophy and myocardial fibrosis are considered common findings of acromegaly-related cardiomyopathy. Histology has revealed interstitial fibrosis is one of the most relevant abnormalities [3]. Different echocardiographic studies have confirmed histological findings, with the authors reporting that cardiac fibrosis is common in acromegaly [3]. Conversely, recent studies using CMR imaging to evaluate patients with active acromegaly found cardiac fibrosis to be totally absent [10, 26] or rare [29, 31], ranging from 0 to 15% of cases. Myocardial T1 mapping and ECV are recent techniques in CMR imaging that allow further tissue characterization. The extra-cellular volume of the myocardium measures the volume fraction of heart tissue that is not taken by cells and reflects interstitial fibrosis, or it can be filled by water; thus, both would potentially increase the ECV [20]. An ECV greater than 30% is suggestive of fibrosis [24]. In our cohort, three patients, although in biochemical remission (2 under medical treatment and 1 surgically cured), had ECV greater than 30%. The proportion of patients with cardiac fibrosis was slightly higher in our cohort than the 0–15% mentioned in previous studies [26, 29, 31], but lower than that reported in echocardiographic studies [26, 29]. Although these discrepancies might reflect the differences in the evaluation methods across the studies, the T1 mapping technique did not show any significant difference between ACRO and CTRLs.

Several echocardiographic studies have evaluated cardiac structure and function in patients with acromegaly and found cardiac hypertrophy, mainly involving the LV [2, 3]. Although echocardiography is common in clinical practice, ultrasound measurement of RV volumes is challenging and the RV is anatomically and functionally different from the LV, therefore LV physiopathology cannot be easily translated to the right heart [32]. It is noteworthy that the RV plays an essential role in determining symptomatic status and prognosis in nearly all cardiovascular disorders. Its response to pathological triggers is a consequence of various combinations of pressure and/or volume overload as well as intrinsic myocardial deficits. Therefore, a comprehensive cardiac assessment should include an accurate evaluation of both ventricles.

Finally, our study confirmed that sex might affect cardiac morpho-functional changes induced by GH-IGF1 excess as recently hypothesized [10, 31]. However, unlike previous studies, we showed that male patients exhibited higher IVS thickness and LVMi compared to females even after adjusting for population age and sex reference ranges, supporting a possible interaction of sexual hormones in cardiac disease progression in acromegaly-related cardiomyopathy, as observed in other cardiovascular diseases [33–35]. In ACRO patients, hypogonadism was present in the 61.5% and 28.6% of males and females, respectively. Although hypogonadal men were adequately replaced on testosterone therapy at study enrolment, we cannot exclude a detrimental impact of male hypogonadism on cardiomyopathy [36].

This study does have limitations. The first is the small study population, although acromegaly is a rare disease and CMR is a complex, expensive, and time-consuming procedure. It should be considered that CMR requires high compliance throughout the examination, potentially affecting patient willingness to perform it for research purposes. However, the number of patients included in our study is in line with other CMR studies in acromegaly. The second is the heterogeneity of the study population since it includes both patients cured after surgery and active patients requiring different medical therapies according to their individual characteristics. However, most of our patients were in biochemical remission. Moreover, it was not possible to obtain data regarding the diagnostic delay since symptom onset. These aspects may have underestimated the cardiac impairment, but we performed subgroup analyses according to disease status, time between initial diagnosis and disease control attainment, and type of medical treatment at the time of study enrolment, permitting us to better stratify cardiac impairment in our population, demonstrating that biventricular cardiac abnormalities may persist even when disease control was achieved. Of note, the limited power of small subgroup comparisons may have resulted in a lack of differences found between subgroups, highlighting the need for further prospective studies with larger cohorts. Finally, our study reported a male predominance, likely due to higher compliance of males than females for research protocols. However, we corrected our results for sex to limit the impact on subgroup analysis.

The pathogenesis of acromegaly-related cardiomyopathy has been proposed to develop after three steps, the earlier of whom is reversible and is characterized by initial cardiac hypertrophy, with increase of heart rate and systolic output. Therefore, to prompt optimal management, the early and reliable detection of cardiac structural and functional abnormalities is mandatory in these patients. We propose a comprehensive characterization of cardiac disease, evaluating both ventricles and myocardial fibrosis by CMR, in patients with acromegaly going beyond the traditional cardiometabolic risk factors and considering the disease status, medical treatments, and the sex-related differences in cardiac damage for a prompt and tailored treatment for both men and women.

## Conclusions

Acromegaly is a rare endocrine disease associated with increased cardiovascular morbidity and mortality, mainly due to congestive heart failure. Our results confirm CMR as a useful tool to detect cardiac abnormalities in acromegaly, even when the disease is adequately controlled, because biventricular structural and functional impairment may persist likely due to a prevalent role of disease-specific pathways triggered by the activation of the GH-IGF1 axis. The results of the current study suggest that CMR may have a place in the cardiac work-up of selected patients for a deeper characterization of cardiac damage, but further studies are needed to clarify which patients may benefit from this approach to implement new strategies of cardiovascular prevention.

## Data Availability

The datasets used and/or analysed during the current study are available from the corresponding author upon reasonable request.
